# Prospective identification of variables as outcomes for treatment (PIVOT): study protocol for a randomised, placebo-controlled trial of hydroxyurea for Ghanaian children and adults with haemoglobin SC disease

**DOI:** 10.1186/s13063-023-07649-7

**Published:** 2023-09-22

**Authors:** Luke R. Smart, Catherine I. Segbefia, Teresa S. Latham, Susan E. Stuber, Kwesi N. Amissah-Arthur, Klenam Dzefi-Tettey, Adam C. Lane, Yvonne A. Dei-Adomakoh, Russell E. Ware

**Affiliations:** 1 Division of Hematology, Cincinnati Children’s Hospital Medical Center, Cincinnati, USA; 2 Department of Pediatrics, University of Cincinnati College of Medicine, Cincinnati, USA; 3 Global Health Center, Cincinnati Children’s Hospital Medical Center, Cincinnati, USA; 4 Department of Child Health, University of Ghana Medical School, Accra, Ghana; 5 Department of Child Health, Korle Bu Teaching Hospital, Accra, Ghana; 6 Ophthalmology Unit, Department of Surgery, University of Ghana Medical School, Accra, Ghana; 7 Ophthalmology Unit, Department of Surgery, Korle Bu Teaching Hospital, Accra, Ghana; 8 Department of Radiology, Korle Bu Teaching Hospital, Accra, Ghana; 9 Department of Haematology, University of Ghana Medical School, Accra, Ghana; 10 Department of Haematology, Korle Bu Teaching Hospital, Accra, Ghana; 11 Ghana Institute of Clinical Genetics, Korle Bu, Accra, Ghana

**Keywords:** Sickle cell disease, Haemoglobin SC disease, Hydroxyurea, Adults, Children

## Abstract

**Background:**

Haemoglobin SC (HbSC) is a common form of sickle cell disease (SCD), especially among individuals of West African ancestry. Persons with HbSC disease suffer from the same clinical complications and reduced quality of life that affect those with sickle cell anaemia (HbSS/Sβ^0^). Retrospective anecdotal data suggest short-term safety and benefits of hydroxyurea for treating HbSC, yet rigorous prospective data are lacking regarding optimal dosing, clinical and laboratory effects, long-term safety and benefits, and appropriate endpoints to monitor. Prospective Investigation of Variables as Outcomes for Treatment (PIVOT) was designed with three aims: (1) to measure the toxicities of hydroxyurea treatment on laboratory parameters, (2) to assess the effects of hydroxyurea treatment on sickle-related clinical and laboratory parameters, and (3) to identify study endpoints suitable for a future definitive phase III trial of hydroxyurea treatment of HbSC disease.

**Methods:**

PIVOT is a randomised, placebo-controlled, double blind clinical trial of hydroxyurea. Approximately 120 children and 120 adults ages 5–50 years with HbSC disease will be enrolled, screened for 2 months, and then randomised 1:1 to once-daily oral hydroxyurea or placebo. Study treatment will be prescribed initially at 20 ± 5 mg/kg/day with an opportunity to escalate the dose twice over the first 6 months. After 12 months of blinded study treatment, all participants will be offered open-label hydroxyurea for up to 4 years. Safety outcomes include treatment-related cytopenias, whole blood viscosity, and adverse events. Efficacy outcomes include a variety of laboratory and clinical parameters over the first 12 months of randomised treatment, including changes in haemoglobin and fetal haemoglobin, intracranial arterial velocities measured by transcranial Doppler ultrasound, cerebral oxygenation using near infrared spectrometry, spleen volume and kidney size by ultrasound, proteinuria, and retinal imaging. Exploratory outcomes include functional erythrocyte analyses with ektacytometry for red blood cell deformability and point-of-sickling, patient-reported outcomes using the PROMIS questionnaire, and 6-min walk test.

**Discussion:**

For children and adults with HbSC disease, PIVOT will determine the safety of hydroxyurea and identify measurable changes in laboratory and clinical parameters, suitable for future prospective testing in a definitive multi-centre phase III clinical trial.

**Trial registration:**

PACTR, PACTR202108893981080. Registered 24 August 2021, https://pactr.samrc.ac.za

## Administrative information

Note: the numbers in curly brackets in this protocol refer to SPIRIT checklist item numbers. The order of the items has been modified to group similar items (see http://www.equator-network.org/reporting-guidelines/spirit-2013-statement-defining-standard-protocol-items-for-clinical-trials/).

**Table:** 

Title {1}	Prospective Identification of Variables as Outcomes for Treatment (PIVOT): Study Protocol for a Randomised, Placebo-controlled Trial of Hydroxyurea for Ghanaian Children and Adults with Haemoglobin SC Disease
Trial registration {2a and 2b}.	PACTR, PACTR202108893981080. Registered 24 August 2021, https://pactr.samrc.ac.za/.
Protocol version {3}	October 20, 2021; Version 1.2
Funding {4}	PIVOT receives financial support including drug donation by Addmedica, Inc., (Paris, France). Addmedica has no role in the design of the study, data collection, results analysis, or manuscript submission.
Author details {5a}	^1^Division of Hematology, Cincinnati Children’s Hospital Medical Center, Cincinnati, USA ^2^Department of Pediatrics, University of Cincinnati College of Medicine, Cincinnati, USA ^3^Global Health Center, Cincinnati Children’s Hospital Medical Center, Cincinnati, USA ^4^Department of Child Health, University of Ghana Medical School, Accra, Ghana ^5^Department of Child Health, Korle Bu Teaching Hospital, Accra, Ghana ^6^Ophthalmology Unit, Department of Surgery, University of Ghana Medical School, Accra, Ghana ^7^Ophthalmology Unit, Department of Surgery, Korle Bu Teaching Hospital, Accra, Ghana ^8^Department of Radiology, Korle Bu Teaching Hospital, Accra, Ghana ^9^Department of Haematology, University of Ghana Medical School, Accra, Ghana ^10^Department of Haematology, Korle Bu Teaching Hospital, Accra, Ghana ^11^Ghana Institute of Clinical Genetics, Korle Bu, Accra, Ghana
Name and contact information for the trial sponsor {5b}	Russell E. Ware, MD PhD Division of Hematology 3333 Burnet Avenue Cincinnati Children’s Hospital Medical Center Cincinnati, OH 45229 Russell.Ware@cchmc.org Phone: 513–803-4597
Role of sponsor {5c}	Cincinnati Children’s Hospital Medical Center (CCHMC) is the sponsor of PIVOT, responsible for the conduct of the study in accordance with all applicable local and international laws and regulations, including without limitation ICHE6 guidelines for Good Clinical Practices.

## Introduction

### Background and rationale {6a}

Sickle cell disease (SCD) is among the world’s most commonly inherited haemoglobin (Hb) disorders. SCD is highly prevalent in sub-Saharan Africa, with more than 300,000 babies born annually, affecting an estimated 1–2% of newborns in some countries [1, 2]. SCD is caused by structural variation in the beta globin component of Hb, and the two most common variants are haemoglobin S (HbS) and haemoglobin C (HbC). Both HbS and HbC result from point mutations affecting the sixth amino acid of beta globin, changing glutamic acid to valine or lysine, respectively. Co-inheritance of two HbS alleles (HbSS) or one HbS allele and one HbC allele (HbSC) results in erythrocytes that are prone to intravascular sickling and cellular dehydration, with a proportion becoming hyperdense cells that promote HbS polymerisation and increase blood viscosity [3].

Individuals with SCD suffer from a wide variety of acute and chronic medical complications that lead to substantial morbidity, poor quality of life, and early mortality [4]. Compared with HbSS, persons with HbSC typically have a less severe form of SCD, yet still suffer from acute and chronic disease complications, including chronic haemolytic anaemia, vaso-occlusive pain, acute chest syndrome, infections, splenomegaly, splenic infarction, gallstones, nephropathy, priapism, and leg ulcers [5, 6]. In HbSC, stroke is relatively less common than in HbSS, presumably due to a higher Hb concentration, while avascular necrosis and retinopathy are more common, due to increased blood viscosity and impaired rheology [7, 8].

In many high-income countries, early identification of SCD through newborn screening and aggressive early interventions, including hydroxyurea therapy have led to marked improvements in both morbidity and mortality [9, 10]. Most efforts to diagnose and manage SCD have focused on HbSS, the most common and severe form [4]. However, HbSC accounts for ~ 25–30% of all diagnoses in the USA [11] and almost 50% of all diagnoses in Ghana.

Research studies funded by the National Heart Lung and Blood Institute (NHLBI) within the National Institutes of Health (NIH) in the USA have collectively documented the safety and efficacy of hydroxyurea therapy for both adults and children with HbSS [12–15]. Laboratory benefits include increased Hb and fetal haemoglobin (HbF) as well as decreased white blood cells (WBC), absolute neutrophil count (ANC), and absolute reticulocyte count (ARC). Clinical benefits include reduced painful vaso-occlusive events, acute chest syndrome, transfusions, and hospitalisations; these effects are consistent and sustainable and optimised when hydroxyurea is titrated to the maximum tolerated dose (MTD) [16–18]. Hydroxyurea therapy is also associated with reduced mortality in HbSS [10, 19–22]. Based on decades of clinical trial evidence, the NHLBI, the American and British Societies of Haematology, and others recommend hydroxyurea treatment for both adults and children with HbSS, beginning at 9 months of age [23–25]. Hydroxyurea has now been approved for the treatment of HbSS by the US Food and Drug Administration (FDA) for adults (in 1997) and children above 2 years (in 2017) as well as the European Medicines Agency (EMA) for all ages (in 2007), but it is not yet approved for the treatment of HbSC disease.

In the NHLBI Evidence-Based Report on Management of SCD, few recommendations refer to HbSC disease, due to lack of high-quality evidence in this large but under-investigated patient population [23]. After the NHLBI evidence-based guidelines were published, a summary of evidence gaps included the “most pressing clinical research needs for disease-modifying therapies” and listed the need to determine the efficacy of hydroxyurea in non-HbSS genotypes, especially HbSC disease [26]. To date, hydroxyurea has not been investigated prospectively as a disease-modifying treatment for HbSC, and it is unknown whether it will have the same salutary effects as for HbSS. Currently, there is only sporadic and anecdotal use of hydroxyurea for HbSC, primarily “clinical drift” from experience with HbSS. For children and adults with HbSC disease, there is a striking lack of evidence about the safety of hydroxyurea treatment, the effects of treatment on a variety of sickle-related clinical and laboratory parameters, and which study endpoints might be suitable for a definitive phase III trial of patients with HbSC disease receiving hydroxyurea therapy. The proposed study will be critical for collecting high-quality evidence that could change practice and potentially help millions of patients with HbSC worldwide.

### Objectives {7}

1. To measure the toxicities of hydroxyurea treatment on laboratory parameters in a large cohort of children and adults with HbSC disease
2. To assess the effects of hydroxyurea treatment on a variety of sickle-related clinical and laboratory parameters in a large cohort of children and adults with HbSC disease
3. To identify which study endpoints are suitable for a phase III trial of patients with HbSC disease receiving hydroxyurea therapy

### Trial design {8}

PIVOT is a prospective randomised double-blind placebo-controlled Phase II clinical trial of hydroxyurea treatment for children and adults with HbSC disease. The trial uses a non-inferiority design framework. An equal number of children (< 18 years old) and adults (≥ 18 years old) as well as male and female participants will be enrolled. Treatment randomisation between hydroxyurea and placebo will occur 1:1. After 12 months of randomised study treatment, haematological toxicity rates will be compared between arms. If there is no evidence of harmful toxicities related to hydroxyurea treatment, all study participants will then be offered open-label hydroxyurea, which will continue until 4 years from the date of first treatment.

## Methods: participants, interventions, and outcomes

### Study setting {9}

PIVOT will be conducted at Korle Bu Teaching Hospital (KBTH), which is a premier referral, consultancy, and tertiary care hospital in Accra, Ghana. Currently, KBTH, which is the third biggest referral centre in Africa, has 2000 beds, 21 clinical and diagnostic departments, and four Centres of Excellence. It has both a Paediatric and an Adult Sickle Cell Clinic (Ghana Institute of Clinical Genetics).

### Eligibility criteria {10}

Potential participants who meet all of the following criteria are eligible for enrolment into the study:

1. Documented HbSC disease
2. Age: ≥ 5.0 and ≤ 50.0 years of age, at the time of enrolment
3. Steady-state laboratory values in the following ranges:
  - Hb 6.0–12 g/dL
  - ARC > 50 × 10^9^/L
  - WBC count > 2.0 × 10^9^/L
  - ANC > 1.0 × 10^9^/L
  - Platelet count > 75 × 10^9^/L
4. Participant, parent, or guardian willing and able to provide informed consent
5. Ability to comply with all study related treatments, evaluations, and follow-up

Potential participants who meet any of the following criteria are disqualified from enrolment in the study:

1. Hydroxyurea treatment currently or within the past 6 months
2.  ≥ 6 blood transfusions within the past 12 months
3.  > 10 hospitalisations in the past 12 months
4. Serum creatinine more than twice the upper limit for age AND ≥ 176.8 μmol/L
5. Any known underlying condition or illness, including chronic pain, co-morbid chronic disease (e.g. tuberculosis, retroviral infection, etc.) which makes study participation ill-advised
6. Use of other therapeutic agents for SCD such as L-glutamine, arginine, crizanlizumab, rivipansel, voxelotor, decitabine, or magnesium currently or within the past 6 months (temporary exclusion)
7. Previous stem cell transplant or other myelosuppressive therapy
8. Inability to take or tolerate daily oral hydroxyurea, including known allergy to hydroxyurea therapy or known malignancy
9. Pregnancy (for post-menarchal females only)
10. Participation in a therapeutic clinical trial or prior disease-modifying treatments currently or in the past 6 months
11. Blood transfusion within the past 2 months (temporary exclusion)

### Who will take informed consent? {26a}

Study nurses and doctors are delegated to obtain informed consent from adults and parents or guardians of children < 18 years old, as well as assent from children 10–17 years old. After detailed discussion of the protocol and the risks and benefits of participation, a copy of the informed consent document (and assent when appropriate) for review and times for questions provided before any forms are signed.

### Additional consent provisions for collection and use of participant data and biological specimens {26b}

Two additional questions will allow participants/guardians to permit or decline: (1) the storage of research specimens for future research and (2) the sharing of research specimens with future collaborators.

## Interventions

### Explanation for the choice of comparators {6b}

There is no current standard therapy recommended for all patients with HbSC disease; therefore, placebo was chosen as the optimal comparison. Two therapies are currently approved by the US FDA and the EMA for treatment of HbSC disease (crizanlizumab and voxelotor), but the trials that led to their approval enrolled very few individuals with HbSC [27, 28], the benefits for HbSC were less certain, and they were approved for treatment of those ≥ 16 years old (crizanlizumab) or ≥ 12 years (voxelotor) at the time this trial was designed. Therefore, neither treatment was deemed an appropriate comparator.

### Intervention description {11a}

Oral hydroxyurea and placebo will be prescribed once daily with instructions to self-administer ideally at a regular time either in the morning or before bedtime. If the time of a scheduled dose is missed, the dose may be administered at any time during the same calendar day. For children incapable of swallowing tablets, the tablets can be chewed or crushed for mixing with water or juice. Dosing will occur with 1000 mg tablets (scored in 250 mg) and 100 mg tablets (scored in 50 mg). Study treatment will be started at a dose of 20.0 ± 5.0 mg/kg/day.

The dose will be escalated up to twice in the first 6 months if all of the following are true: ANC > 3.0 × 10^9^/L, Hb > 7.0 g/dL, ARC > 80 × 10^9^/L, platelets > 100 × 10^9^/L. Dosing flexibility is permitted to allow for rounding to available tablet strengths. A dosing calculator will provide a recommended dose. The maximum daily dose of study treatment will not exceed 2000 mg or 35 mg/kg/day without prior discussion and approval from the PIVOT Medical Coordinating Centre (MCC). Minor dose adjustments will be made as necessary to accommodate any changes in a participant’s weight. In the event of splenomegaly and hypersplenism, dose adjustments may be modified after consultation with the MCC.

### Criteria for discontinuing or modifying allocated interventions {11b}

Study treatment will be temporarily discontinued for haematological toxicity defined as any of the following: ANC < 1.0 × 10^9^/L; Hb < 4.0 g/dL regardless of ARC; Hb < 6.0 g/dL with an ARC < 100 × 10^9^/L; ARC < 80 × 10^9^/L with an Hb < 7.0 g/dL, or platelet count < 80 × 10^9^/L. If recovery occurs after 1 week, study treatment may resume at the previous dose. If toxicity persists for > 2 weeks or occurs twice within a 3-month period in the absence of another potential cause, study treatment will be held until recovery and then reduced by 2.5 mg/kg/day. In certain limited clinical circumstances, laboratory parameters below these haematological thresholds do not represent true marrow suppression and dose-limiting toxicity, and the investigators and MCC after discussion will have discretion whether or not to reduce the daily treatment dose.

In addition, if the Hb exceeds 12 g/dL (with ≥ 1.0 g/dL increase from the baseline value); then, this will be recorded as a laboratory toxicity. If the study participant has symptoms of hyperviscosity such as dizziness or headache, then a phlebotomy of 5–10 mL/kg can be performed, with full-volume replacement of IV normal saline to prevent hypovolaemia, and study treatment will continue.

If a female becomes pregnant, treatment will be discontinued for the duration of the pregnancy, but the participant will remain in the trial. Investigators may discontinue treatment for any participant at their discretion if, in their professional opinion, the participant’s health, safety, and/or well-being is threatened by continued participation in the study.

### Strategies to improve adherence to interventions {11c}

Participants will be reminded about the importance of consistent once daily dosing at each study visit. Participants will be instructed to return unused medication at each study visit, and the number of unused tablets will be counted and recorded. Counselling will be provided to those who report low adherence.

### Relevant concomitant care permitted or prohibited during the trial {11d}

All care is permissible, except for the medications mentioned in the exclusion criteria.

### Provisions for post-trial care {30}

Participants will be enrolled from the sickle cell clinics where they usually attend. Their care throughout the trial will be overseen by their usual clinical team in accordance with the national guidelines and local clinical practices. After the initial 12 months of randomised treatment, all participants will be invited to continue open label treatment until 4 years from the first treatment initiation. Any incidental findings will be shared with their clinical team. Post-trial care will be provided by the local clinical team. Clinical trial insurance will provide compensation to those who suffer harm from trial participation.

### Outcomes {12}

The following outcomes will be measured after 12 months of therapy:

1. Cumulative incidence of haematological toxicities (primary outcome)
2. Cumulative incidence of laboratory and clinical adverse events ≥ grade 2 and serious adverse events (SAEs), graded according to the Common Terminology Criteria for Adverse Events (CTCAE) version 4.0, except for minor adjustments to laboratory grading due to the underlying nature of SCD as described in the “Adverse event reporting and harms {22}” section
3. Change from baseline in a variety of laboratory and clinical parameters
  - Cerebral Hb oxygen saturation
  - Three-dimensional splenic volume
  - Intracerebral arterial blood flow velocity
  - Grade of retinopathy
  - Whole blood viscosity
  - Erythrocyte deformability
  - Quality of life
4. Cumulative incidence of pain, acute chest syndrome, acute splenic sequestration, sepsis, malaria, stroke, and death over 12 months of therapy
5. Cumulative incidence of blood transfusions and hospitalisations

### Participant timeline {13}

**Fig. 1:**
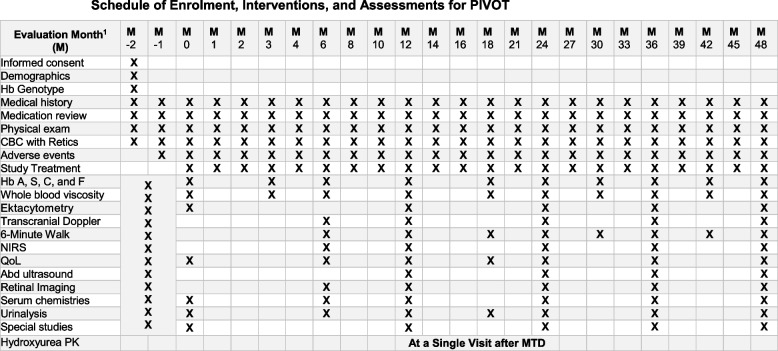
Schedule of enrolment, interventions, and assessments for PIVOT. Superscript digit one (.^1^) indicates the following: visits may occur within a window of+/- 7 days during months 0-24 and +/-14 days during months 24-48

After written informed consent is obtained, participants will complete at least 2 months of screening visits before randomisation. After randomisation, participants will receive either oral hydroxyurea or placebo for 12 months, followed by an opportunity for subsequent open label treatment that will continue up to 48 months from the time of first participant randomisation. See Fig. 1 for schedule of events.

### Sample size {14}

The primary study hypothesis is that hydroxyurea treatment for children and adults with HbSC disease will not cause excess haematological toxicities. We shall use a non-inferiority study design to compare toxicity rates between the treatment arms. In the US experience, [29] laboratory toxicities were identified in ~ 20% of the HbSC patients receiving hydroxyurea, but these were retrospective data and the dose was not escalated to MTD. In the PIVOT trial, we estimate a cumulative haematological toxicity rate of ~ 20% in the placebo arm and allow a 15% margin for non-inferiority in the hydroxyurea arm. Based on these assumptions, we calculate that 176 study participants are needed with an overall 80% power. The enrolment target will be 240 patients (50% children, 50% adults). This sample size will allow 10% screen fail and 8% drop out in year 1, leaving 100 evaluable participants in each age cohort who will receive placebo-controlled study treatment for 12 months. We estimate ≤ 25% dropout rate over the 4-year span of the study. We will not enrol additional patients if the dropout rate exceeds the estimated rate.

### Recruitment {15}

The study will be discussed with patients at their regularly scheduled visits to the adult and paediatric sickle cell clinics, and printed informational fliers will be distributed to patients and their families to sensitise them to the trial.

## Assignment of interventions: allocation

### Sequence generation {16a}

Study participants will be randomised to one of two treatment categories (hydroxyurea or placebo) at an approximately 1 to 1 ratio. Randomisation will be stratified at the two sites (adult and paediatric) to achieve approximate balance with respect to age at enrolment. Participants will be randomly assigned to a blinded treatment arm using the randomisation module in REDCap Cloud, a secure, web-based, 21 CRF Part 11-compliant electronic data capture system. The data coordinating centre programmed REDCap to randomise in blocks of 8 participants and to stratify by age (above or below 18 years at time of consent), and the allocation sequence is generated by the randomisation software in REDCap.

### Concealment mechanism {16b}

The study pharmacist will know whether the participant is randomised to group A or group B but will also be blinded and not know which group is active drug or placebo. The tablets for group A and group B will have an identical appearance.

### Implementation {16c}

After completion of screening visits, participants’ clinical history and laboratory results will be reviewed by the MCC at CCHMC. The MCC will inform the data coordinating centre (DCC) about appropriateness for randomisation and the DCC will then notify the clinical site regarding readiness to randomise. The clinical site will schedule a study visit at which randomisation will occur. At the randomisation visit, updated labs will be collected and reviewed by the study physician. If the labs are appropriate for treatment initiation, the study physician will inform the pharmacy that the participant is ready for randomisation. The clinical pharmacist will access the secure interactive web randomisation system, randomise the participant to either group A or group B using the REDCap Cloud randomisation module, and dispense the correct treatment.

## Assignment of interventions: blinding

### Who will be blinded {17a}

All investigators, the site study team, the participants, the parents, outcome assessors, and data analysts will be blinded to the study treatment assignment. The participant’s study ID and treatment allocation will be recorded in a blinded list, to which only the CCHMC DCC director and lead database administrator will have access until the study is completed or stopping rules are reached.

Since hydroxyurea at MTD causes predictable changes to the complete blood count (CBC) such as increased mean corpuscular volume (MCV) and lower WBC and ANC, all investigators will agree to not view the routine clinic CBC results or assess laboratory trends; instead, another designated healthcare provider will examine the local lab results for possible toxicities and communicate with the local study pharmacist about appropriate dosing including escalation.

### Procedure for unblinding if needed {17b}

If an acute safety concern arises that requires a study participant’s randomised treatment assignment to be unblinded, the site will immediately generate an email to the principal investigators (PIs) and the DCC director with complete details of the situation. If the decision to unblind is made, the DCC director and lead database administrator will determine the allocation code according to the randomisation file and disclose the participant’s treatment assignment to the site PI or designee. The allocation will not be disclosed to other study personnel, monitors, nor the study participant’s family. There will not be verbal disclosure of the code at any time. Details of the time, circumstances, and reason for emergency unblinding are to be documented in the participant’s medical records and any relevant case report forms (CRFs).

## Data collection and management

### Plans for assessment and collection of outcomes {18a}

Sickle-related clinical events will be collected during interval study visits through both patient and family reports and review of medical records. Physical examination will be performed by study clinicians experienced with SCD. A CBC will be performed using a haematology analyser with a five-part leukocyte differential and automated reticulocyte count. Whole blood viscosity will be performed using a Brookfield Ametek rheometer. Erythrocyte deformability and point-of-sickling will be assessed using an RR Mechantronics Lorrca MaxSis. Hb types will be distinguished and quantitated using high-performance liquid chromatography (HPLC). The 6-min walk test will be assessed by study clinicians after completion of training and demonstration of competency. Cerebral regional tissue Hb oxygen saturation will be assessed using near infrared spectroscopy bilaterally. Spleen volume and kidney length will be assessed using ultrasound performed by a consultant radiologist. Intracerebral arterial velocities will be assessed using transcranial Doppler (TCD) ultrasound performed by certified examiners who have completed a training course and submitted an adequate number of high-quality exams. Retinopathy will be assessed using retinal photography and optical coherence tomography, with angiography for positive cases, performed by experienced ophthalmology technicians and interpreted by a consultant ophthalmologist. Quality of life will be assessed using the PROMIS questionnaire.

### Plans to promote participant retention and complete follow-up {18b}

Participants will be reimbursed a small amount for transportation and time spent at study visits. To enhance participant retention, more than one mobile phone number will be obtained for each patient and family. Permission will be obtained to contact a close friend or neighbour in the event that their mobile phone is out of service. They will be sent SMS text reminders prior to their follow-up visits.

### Data management {19}

Study data will be collected and managed using the REDCap Cloud™ electronic data capture system. REDCap Cloud™ is a secure 21 CFR Part 11-compliant web-based application designed to support data capture for research studies, providing (1) an intuitive interface for validated data entry, (2) audit trails for tracking data manipulation and export procedures, (3) automated export procedures for seamless data downloads to common statistical packages, and (4) validated electronic signatures. The REDCap Cloud™ system supports a secure web application designed exclusively to support data capture for research studies, with a secure internet-based connection that uses low bandwidth, suitable for research in low- and middle-income countries.

Data will be entered into the electronic data record directly from the clinical site. Electronic data will be reviewed for accuracy and completion by research staff. Programmes will be created to run logical and missing value error checks on the data files. Audit reports will result from these programmes and will be used to clean the data. Quality control analyses are performed on the data at standard time intervals. Outliers will be reviewed, resolved, and the database updated as necessary.

The DCC will remotely monitor data accuracy and completeness and also be responsible for on-site monitoring. The DCC will be responsible for maintaining the data centrally, randomisation, maintaining the blind, monitoring enrolment and completeness of data, and generating queries to notify sites of incomplete data. In addition, the DCC will be responsible for statistical analysis, including summary reports for MCC review and performing on-site monitoring visits to compare submitted data with source documents. The DCC will also prepare a semi-annual report for the independent data safety monitoring board (DSMB). The study site is responsible for reporting AEs centrally to the DCC via electronic data entry.

Clinical site monitoring is conducted to ensure that the rights and well-being of trial participants are protected, that the reported trial data are accurate, complete, and verifiable, and that the conduct of the trial is in compliance with the currently approved protocol/amendment(s), with International Conference on Harmonisation Good Clinical Practice (ICH GCP), and with applicable regulatory requirement(s). A local monitor will be employed for on-site trial monitoring following the data safety monitoring plan (DSMP). Additional remote database and on-site monitoring will be conducted by the MCC and DCC as assigned by the international PI.

### Confidentiality {27}

Each participant will be given a study ID number, which will allow de-identified information to be collected without the participant’s name, including clinical information and laboratory results. No information concerning the study or the data will be released to any unauthorised third party without prior written approval of the sponsor. All research activities will be conducted in as private a setting as possible. Study participant contact information will be securely stored at each clinical site for internal use during the study. At the end of the study, all records will be securely maintained for as long a period as dictated by the reviewing institutional review board (IRB), institutional policies, regulatory authority, or sponsor requirements.

### Plans for collection, laboratory evaluation, and storage of biological specimens for genetic or molecular analysis in this trial/future use {33}

Peripheral blood will be collected and placed onto a Whatman FTA card and into a PaxGene tube for DNA preservation, and genomic DNA will be purified later using standard laboratory techniques. These samples will be analysed using a variety of laboratory methods such PCR, Sanger sequencing, and next generation sequencing to identify genetic variations that could influence the clinical phenotype in HbSC disease or to modify hydroxyurea response. If participants agree during the informed consent, specimens will be stored for future research.

## Statistical methods

### Statistical methods for primary and secondary outcomes {20a}

The lab and clinical parameters will be analysed either as continuous or categorical variables. Event rates for toxicities and clinical complications will be compared to baseline after 6 and 12 months of blinded study treatment, using non-inferiority methods.

### Interim analyses {21b}

A formal interim analysis is not planned, but the DSMB will receive a summary of accrued data every 6 months. The DSMB will recommend either continuation or termination of the study at each meeting. Termination may be suggested at any time and reasons for early termination include, but are not limited to, SAEs affecting more than one third of the study participants enrolled.

### Methods for additional analyses (e.g. subgroup analyses) {20b}

Subgroup analysis will be performed to analyse the benefit of hydroxyurea in participants with high versus low incidence of pain episodes and by baseline clinical and laboratory factors that indicate more and less severe disease.

### Methods in analysis to handle protocol non-adherence and any statistical methods to handle missing data {20c}

All study data collected from eligible participants will be used in the analysis of primary and secondary outcomes. For participants who withdraw prior to study completion, all data points will be included in analysis up to and including the time of study withdrawal. Missing data related to primary endpoints will not be imputed.

### Plans to give access to the full protocol, participant-level data, and statistical code {31c}

De-identified data will be made available to all investigators whose proposed use of data has been approved by an internal review committee identified for this purpose and with a signed data-sharing agreement between all parties.

## Oversight and monitoring

### Composition of the coordinating centre and trial steering committee {5d}

The primary PIVOT clinical site will be led by two local PIs specialised in adult haematology and paediatric haematology respectively. Both of these local PI’s have investigative research teams at KBTH. The PIVOT study team in KBTH will also include a study coordinator, study physicians, nurses, pharmacists, and research assistants.

The PIVOT trial will be guided by both the MCC and DCC located at CCHMC. The MCC will be led by the international PI and a co-investigator. The co-investigator will be the primary clinical coordinator to assist the site with clinical questions, dosing issues, AEs, and other questions related to interpretation of the protocol. The DCC is led by the international PI and assisted by a team of experienced personnel who have substantial expertise in multicentre clinical trials, online electronic data capture, and hybrid monitoring of data quality and completeness. The whole team (KBTH and CCHMC) will meet monthly via video conference, and the local site coordinators will meet with the CCHMC coordinators on a separate monthly call. An annual in-person meeting will occur along with an on-site sponsor monitoring visit conducted by MCC and DCC leadership.

### Composition of the data monitoring committee, its role and reporting structure {21a}

An independent DSMB has been formed to provide safety and oversight for the study. The DSMB is composed of international experts in SCD, both paediatric and adult, as well as clinical trial design and patient advocacy. The DSMB will convene every 6 months to review accrued data summaries prepared by the DCC. The DSMB will make recommendations to the MCC for either continuation or termination of the study at each meeting. Termination may be suggested at any time and reasons for early termination include, but are not limited to, SAEs affecting more than one third of the study participants enrolled.

### Adverse event reporting and harms {22}

To document medication safety, AEs and SAEs will be collected and reported to the MCC and DCC using data collection forms. AE reporting will utilise the CTCAE Version 4.0, where all AE are categorised by organ system and graded by severity. Only AE grade 2 and above including all SAE will be reported. Table 1 lists exceptions to the CTCAE version 4.0 guidelines that will be used for AE grading of selected laboratory parameters in the PIVOT study.

**Table 1:** List of laboratory exceptions to the CTCAE, version 4.0, grading guidelines

Parameter	Grade 2	Grade 3	Grade 4
Hb (g/dL)	5.0–6.0	4.0–4.9	< 4.0
WBC (× 10^9^/L)	1.0–1.999	0.5–0.999	< 0.5
ANC (× 10^9^/L)	0.5–0.999	0.2–0.499	< 0.2
Platelets (× 10^9^/L)	50–79	20–49	< 20
Total bilirubin (μmol/L)	85.5–171	171.1–342	> 342
AST (IU/L)	150–300	301–1000	> 1000
ALT (IU/L)	150–300	301–1000	> 1000
Creatinine (μmol/L)	Doubling of baseline serum creatinine and value ≥ 88.4 μmol/L	141.44–176.8	> 176.8
ARC (× 10^9^/L) with Hb < 7.0 g/dL	50–80	10–49	< 10

Several complications are expected to occur in PIVOT because of the underlying disease process of HbSC (e.g. haemolytic anaemia, acute painful episodes, etc.) and the known side effect profile of hydroxyurea. A list of many expected and potentially serious AE, as well as exceptions to the CTCAE, is included in Tables 2 and 3. A PIVOT unexpected AE is defined as any AE or SAE that is not usually associated with either the underlying disease process of HbSC or treatment with hydroxyurea. The PI will use clinical judgement along with consensus knowledge about SCD in determining event expectedness. Hydroxyurea-related complications will be reviewed by the PIVOT PI or MCC representative to determine the ability to continue therapy.

**Table 2:** List of expected and potentially serious adverse events in all PIVOT participants with HbSC related to sickle cell disease

• Abnormal TCD velocities	• Fever without source	• Pain, non-specific
• Acute chest syndrome	• Haematuria	• Pain, severe abdominal
• Adenotonsillar disease	• Haemolysis	• Pain, sternal or rib
• Albuminuria	• Haemorrhagic Stroke	• Pneumonia
• Amenorrhea	• Hand-foot syndrome/dactylitis	• Priapism
• Anaemia (severe)	• Headache	• Proteinuria
• Aplastic crisis	• Hemiplegia	• Pulmonary embolism
• Arthralgia	• Hepatic sequestration	• Pulmonary hypertension
• Avascular necrosis of hip/shoulder	• Hepatomegaly	• Pulmonary infiltrate on chest x-ray
• Bacteraemia	• Hospitalisation > 24 h	• Pyelonephritis
• Bone infarction	• Hyperbilirubinemia	• Recurrent wheezing episodes
• Cardiac arrhythmia	• Hypersplenism	• Renal failure
• Cardiomegaly	• Hypertension	• Renal insufficiency
• Cerebrovascular accident	• Hypocalcaemia	• Renal papillary necrosis
• Cholecystitis	• Hyposthenuria	• Reticulocytopenia
• Cholelithiasis	• Hypotension	• Reticulocytosis
• Cognitive dysfunction	• Hypoxemia (PO2 < 65 mm Hg)	• Retinal haemorrhage
• Constipation	• Ileus	• Retinopathy
• Cranial nerve palsy	• Infection, line	• Rhabdomyolysis
• Death	• Infection, other bacterial	• Seizure
• Decreased lung function	• Infection, pneumococcal	• Septicaemia
• Decreased renal function	• Infection, viral	• Silent infarct
• Delayed growth/puberty	• Jaundice	• Skin ulcer
• Depression	• Leukocytosis	• Sleep apnoea
• Dizziness	• Meningitis	• Splenic sequestration
• Electrolyte imbalance	• Nephropathy	• Splenomegaly
• Elevated serum transaminases	• Osteomyelitis	• Stroke
• Elevated TCD velocities	• Pain, back	• TIA
• Elevated urinary urobilinogen	• Pain, chest	• Tonsillar hypertrophy
• Empyema	• Pain, joint	• Transfusion, unanticipated
• Fever with infection	• Pain, long bone	• Vaso-occlusive pain

**Table 3:** List of expected and potentially serious adverse events in PIVOT associated with hydroxyurea therapy

• Allergic reaction	• Hypersplenism	• Pancytopenia
• Anaemia	• Increased ALT	• Rash
• Anorexia	• Increased creatinine	• Reticulocytopenia
• Constipation	• Leukopenia	• Skin ulcers/gangrene
• Diarrhoea	• Nail/skin hyperpigmentation	• Splenomegaly
• Gastritis	• Nausea	• Thrombocytopenia
• Hair loss	• Neutropenia	• Vomiting
• Increase in at least one of the following laboratory parameters: MCV, MCH, MCHC, nucleated RBC, Hb electrophoresis (%S and %F)		
• Decrease in at least one of the following laboratory parameters: ARC, % reticulocytes, RBC, WBC, ANC, platelet count, haematocrit, Hb electrophoresis %A, total bilirubin, LDH, and ferritin		

An SAE is an AE occurring during any study phase (screening, treatment or follow-up) that fulfils one or more of the following criteria:

1. Results in death
2. Is immediately life-threatening
3. Requires in-patient hospitalisation > 7 days or prolongs an existing hospitalisation
4. Results in persistent or significant disability/incapacity
5. Is a congenital abnormality or birth defect
6. Is an important medical event that may jeopardise the subject or may require medical intervention to prevent one of the outcomes listed above

All SAEs will be reported to the KBTH IRB and Ghana FDA, per their reporting requirements. Additionally, SAEs will be promptly reported to the MCC and DCC safety officer. If an ongoing SAE changes in its intensity or relationship to the investigational product, a follow-up SAE report will be sent to the KBTH IRB, Ghana FDA, and MCC. All SAEs will be followed until resolution or stabilisation. SAE reporting will be completed using the PIVOT SAE CRF available in the study database.

### Frequency and plans for auditing trial conduct {23}

Clinical site monitoring will be conducted to ensure that the rights and well-being of trial participants are protected, that the reported trial data are accurate, complete, and verifiable, and that the conduct of the trial is compliant with the currently approved protocol/amendment(s), with ICH GCP, and with applicable regulatory requirement(s). A local monitor will be employed for on-site trial monitoring following the DSMP and Ghanaian regulatory requirements. Remote database monitoring will occur at least monthly by the DCC, and annual on-site sponsor monitoring will occur in addition to visits performed by the locally-assigned monitor.

### Plans for communicating important protocol amendments to relevant parties (e.g. trial participants, ethical committees) {25}

Any protocol amendments will be agreed upon by the investigators and submitted for approval to the IRB and Ghana FDA for approval before implementation. The trial registry will be updated after ethical/regulatory approval has been obtained. Trial participants will be informed of any changes that affect their involvement or treatment, and if necessary, they will be re-consented for ongoing participation.

### Dissemination plans {31a}

At the end of the trial, a main outcome manuscript will be submitted to a peer reviewed medical journal and the trial results will be presented at an international meeting. Other results generated from the trial that are not published in the main outcome manuscript will be submitted for publication in peer-reviewed medical journals and presented at local, national, regional, and international scientific meetings. Addmedica has no role in the analysis of results or decision to publish results and will not write, review, or comment on manuscripts.

## Discussion

PIVOT is the first randomised controlled phase 2 trial of hydroxyurea for the treatment of HbSC disease. Prospective therapeutic trials for treatment of HbSC disease are sorely needed, and hydroxyurea is a natural candidate given the extensive experience success in treatment for HbSS. This will be the largest prospective description of children or adults with HbSC disease treated with hydroxyurea, enrolling 120 children and 120 adults who will be randomised to active drug or placebo for 12 months before an open-enrolment phase with prolonged follow-up to describe the long-term safety and effectiveness of hydroxyurea.

Hydroxyurea is a logical and intuitive treatment for HbSC, due to its known potency to induce HbF and inhibit sickle polymerisation, as well as its low cost and ease of oral administration. The importance of HbF in HbSC disease remains undefined, though HbF is known to delay both HbS polymerisation and HbC crystallisation [3]. PIVOT will simultaneously evaluate the laboratory and clinical responses to hydroxyurea among both paediatric and adult populations. Despite the plethora of data regarding hydroxyurea treatment for HbSS, there are relatively few published studies for patients with HbSC, and much less is known about its benefits and risks for this population, especially in adults.

The first descriptions of haematological response to hydroxyurea in HbSC disease were published 25 years ago [30] when it was noted in a small case series (6 patients) that low dose hydroxyurea (10–20 mg/kg/day) increased the Hb, MCV, and HbF while decreasing neutrophils and reticulocytes. This was soon followed by several other small case series that confirmed haematological changes with low dose [31] and both haematological and clinical improvement with escalated dosing [32, 33]. The largest retrospective analysis was a multi-centre survey of US-based academic haematologists that described hydroxyurea effects on HbSC among 133 children, teens, and young adults treated at 23 academic centres across the USA. Approximately 5% of the patients with HbSC were receiving hydroxyurea; the median age at treatment initiation was 15 years; and the most common clinical indication was vaso-occlusive pain and/or acute chest syndrome. The number of pain episodes decreased significantly after 12 months of treatment. The reduction in vaso-occlusive pain was 38%, similar to adults with HbSS (44%) or children with HbSS (52%) [13, 15], but the retrospective and open-label nature of the data left several questions unanswered. The hydroxyurea dosing was highly variable, with 20% of treated patients receiving < 15 mg/kg/day while another 20% was taking > 25 mg/kg/day. There appeared to be a greater reduction in painful episodes in those who started on ≥ 20 mg/kg/day in children ≥ 15 years old, but no difference in those < 15 years old. Lab toxicities requiring a dose hold or adjustment occurred in 20% of treated patients, usually due to neutropenia and/or thrombocytopenia. Only one patient reported increased pain in association with a higher Hb level. There were no statistically significant changes in the Hb level after 12 months of treatment, and treatment was associated with modest increases in MCV (+ 11 fL) and HbF (+ 3%), and mild decreases in WBC and ANC [29]. In contrast, PIVOT will initiate blinded study treatment at 20 mg/kg/day in all participants with two opportunities to escalate the dose during the first 6 months of treatment. Dosing toxicities in previous clinical trials of hydroxyurea for HbSS disease in Africa have not revealed a significant increase in cytopenias with fixed or escalated dosing [34–36].

Hydroxyurea is a prime candidate for treatment of HbSC disease because it is relatively inexpensive, widely available, and already used extensively for treatment of HbSS disease in both high-income countries and sub-Saharan Africa. This trial will be conducted in Ghana where the burden of HbSC disease is arguably the highest in the world, and effective long-term treatment is especially needed. Ghana is an ideal location for several reasons: (1) there is a large amount of HbS and HbC in the population, so HbSC is a common form of SCD; (2) the KBTH programmes are established and have well-known leaders in the field; (3) hydroxyurea is now becoming a more common treatment for HbSS in Ghana. This growing familiarity with hydroxyurea makes Ghana an attractive place to conduct additional research studies on hydroxyurea, since the Ghana FDA has approved the medication for HbSS and investigators and families are more aware of the treatment benefits.

The study will be conducted across a large medical campus and involves two separate clinics and a separate research laboratory that will allow collection of a variety of exploratory outcomes. The effect of treatment-related changes in the Hb on the whole blood viscosity will be measured on location with fresh blood and will be correlated to any clinical complications, meeting an important safety aim. Retinopathy is a particularly common complication of HbSC disease possibly because of the density and increased viscosity of the erythrocyte containing both HbS and HbC. We have the first opportunity to test the effect of hydroxyurea on retinopathy in a randomised fashion across age spans and with advanced ophthalmological evaluation. RBC deformability may be a more immediate target that demonstrates clinical improvement over the short term, and the site has been equipped with an ektacytometer and viscometer to capture functional aspects of the RBC before and after hydroxyurea treatment.

Our trial has some potential limitations. The size of the trial may not provide the power needed to demonstrate efficacy if the clinical gains are less prominent over the first year of randomised treatment. We have attempted to provide optimal benefit by starting at a moderate weight-based dose (20 mg/kg/day) and allowing for dose escalation if haematological parameters permit. The optimal clinical outcomes for a trial of HbSC disease are unknown. Many of the clinical morbidities occur later in life and more slowly than in HbSS disease. Some of the most prominent components of disease (e.g. chronic pain from AVN) may not respond quickly enough or be reversible enough to demonstrate efficacy in 12 months.

In summary, PIVOT is the first randomised placebo-controlled blinded trial to provide prospective data regarding the safety of hydroxyurea, the appropriate dose range, and the clinical effects in HbSC. PIVOT will provide invaluable details regarding a wide range of clinical and laboratory outcomes that will not only be essential for the design a future definitive phase 3 clinical trial but will also have immediate clinical implications for the large number of people living with HbSC in Ghana and around the world. Instead of anecdotal usage offered only to patients with severe symptoms or potentially irreversible organ damage, hydroxyurea could emerge as an important treatment option before the onset of acute and chronic clinical complications. If effective and safe as disease modifier, hydroxyurea could be an inexpensive and accessible option that changes the lives of millions of individuals with HbSC disease.

## Trial status

The current PIVOT protocol at the time of manuscript submission is Version 1.2, written on October 20, 2021. Recruitment for the study began on 13 April 2022, and it is estimated that recruitment will finish on or around 30 June 2023.

## Data Availability

Requests for de-identified data may be directed to the PI and must include an IRB-approved proposal. De-identified data will be made available following approval by an internal review committee and execution of a data-sharing agreement between all parties.

## Abbreviations

AE: Adverse event
ALT: Alanine aminotransferase
ANC: Absolute neutrophil count
ARC: Absolute reticulocyte count
AST: Aspartate aminotransferase
CBC: Complete blood count
CCHMC: Cincinnati Children’s Hospital Medical Center
CTCAE: Common Terminology Criteria for Adverse Events
CRF: Case report form
DCC: Data coordinating centre
DSMB: Data safety monitoring board
DSMP: Data safety monitoring plan
EMA: European Medicines Agency
FDA: Food and Drug Administration
Hb: Haemoglobin
HbC: Haemoglobin C
HbF: Haemoglobin F, fetal haemoglobin
HbS: Haemoglobin S
HbSβ^0^: Haemoglobin S-beta thalassemia zero
HbSC: Haemoglobin SC
HbSS: Haemoglobin SS
HPLC: High-performance liquid chromatography
ICH GCP: International conference on harmonization good clinical practice
IRB: Institutional review board
KBTH: Korle Bu Teaching Hospital
LDH: Lactate dehydrogenase
MCC: Medical coordinating centre
MCV: Mean corpuscular volume
MTD: Maximum tolerated dose
NHLBI: National Heart Lung and Blood Institute
NIH: National Institutes of Health
PI: Principal investigator
PIVOT: Prospective Identification of Variables as Outcomes for Treatment
SAE: Serious adverse event
SCD: Sickle cell disease
SPIRIT: Standard protocol items: recommendations for interventional trials
TCD: Transcranial Doppler ultrasound
WBC: White blood cell

## References

[1] World Health Organization (2010). Regional Office for Africa. Sickle-cell disease: a strategy for the WHO African Region.

[2] Piel RB, Patil AP, Howes RE (2013). Global epidemiology of sickle haemoglobin in neonates: a contemporary geostatistical model-based map and population estimates. Lancet.

[3] Nagel RL, Fabry ME, Steinberg MH (2003). The paradox of hemoglobin SC disease. Blood Rev.

[4] Ware RE, de Montalembert M, Tshilolo L, Abboud MR (2017). Sickle cell disease. Lancet.

[5] Powars DR, Hiti A, Ramicone E, Johnson C, Chan L (2002). Outcome in hemoglobin SC disease: a four-decade observational study of clinical, hematologic, and genetic factors. Am J Hematol.

[6] Gualandro SFM, Fonseca GHH, Yokomizo IK, Gualandro DM, Suganuma LM (2015). Cohort study of adult patients with haemoglobin SC disease: clinical characteristics and predictors of mortality. Br J Haematol.

[7] Lemonne N, Lamarre Y, Romana M (2014). Impaired blood rheology plays a role in the chronic disorders associated with sickle cell-hemoglobin C disease. Haematologica.

[8] Pecker LH, Schaefer BA, Luchtman-Jones L (2017). Knowledge insufficient: the management of haemoglobin SC disease. Br J Haematol.

[9] Quinn CT, Rogers ZR, McCavitt TL, Buchanan GR (2010). Improved survival of children and adolescents with sickle cell disease. Blood.

[10] Lê PQ, Gulbis B, Dedeken L (2015). Survival among children and adults with sickle cell disease in Belgium: Benefit from hydroxyurea treatment. Pediatr Blood Cancer.

[11] Therrell BL, Lloyd-Puryear MA, Eckman JR, Mann MY (2015). Newborn screening for sickle cell diseases in the United States: a review of data spanning 2 decades. Semin Perinatol.

[12] Charache S, Dover GJ, Moore RD (1992). Hydroxyurea: effects on hemoglobin F production in patients with sickle cell anemia. Blood.

[13] Charache S, Terrin ML, Moore RD (1995). Effect of hydroxyurea on the frequency of painful crises in sickle cell anemia. N Engl J Med.

[14] Kinney TR, Helms RW, O’Branski EE (1999). Safety of hydroxyurea in children with sickle cell anemia: results of the HUG-KIDS study, a phase I/II trial Pediatric Hydroxyurea Group. Blood.

[15] Wang WC, Ware RE, Miller ST (2011). Hydroxycarbamide in very young children with sickle-cell anaemia: a multicentre, randomised, controlled trial (BABY HUG). Lancet.

[16] Ware RE (2010). How I use hydroxyurea to treat young patients with sickle cell anemia. Blood.

[17] McGann PT, Ware RE (2015). Hydroxyurea therapy for sickle cell anemia. Expert Opin Drug Safe.

[18] Ware RE (2015). Optimizing hydroxyurea therapy for sickle cell anemia. Hematology Am Soc Hematol Educ Program.

[19] Steinberg MH, McCarthy WF, Castro O (2010). The risks and benefits of longterm use of hydroxyurea in sickle cell anemia: a 17.5 year follow-up. Am J Hematol..

[20] Voskaridou E, Christoulas D, Bilalis A (2010). The effect of prolonged administration of hydroxyurea on morbidity and mortality in adult patients with sickle cell syndromes: results of a 17-year, single-center trial (LaSHS). Blood.

[21] Lobo CL, Pinto JF, Nascimento EM, Moura PG, Cardoso GP, Hankins JS (2013). The effect of hydroxycarbamide therapy on survival of children with sickle cell disease. Br J Haematol.

[22] Karacaoglu PK, Asma S, Korur A (2016). East Mediterranean region sickle cell disease mortality trial: retrospective multicenter cohort analysis of 735 patients. Ann Hematol.

[23] Yawn BP, Buchanan GR, Afenyi-Annan AN (2014). Management of sickle cell disease: summary of the 2014 evidence- based report by expert panel members. JAMA.

[24] Qureshi A, Kaya B, Pancham S (2018). Guidelines for the use of hydroxycarbamide in children and adults with sickle cell disease: a British Society for Haematology Guideline. Br J Haematol.

[25] United Republic of Tanzania Ministry of Health (2020). Community Development, Gender.

[26] Savage WJ, Buchanan GR, Yawn BP (2015). Evidence gaps in the management of sickle cell disease: a summary of needed research. Am J Hematol.

[27] Ataga KI, Kutlar A, Kanter J (2017). Crizanlizumab for the prevention of pain crises in sickle cell disease. N Engl J Med.

[28] Vichinsky E, Hoppe CC, Ataga KI (2019). A phase 3 randomized trial of voxelotor in sickle cell disease. N Engl J Med.

[29] Luchtman-Jones L, Pressel S, Hilliard L (2016). Effects of hydroxyurea treatment for patients with hemoglobin SC disease. Am J Hematol.

[30] Steinberg MH, Nagel RL, Brugnara C (1997). Cellular effects of hydroxyurea in HbSC disease. Br J Haematol.

[31] Iyer R, Baliga R, Nagel RL (2000). Maximum urine concentrating ability in children with HbSC disease: effects of hydroxyurea. Am J Hematol.

[32] Miller MK, Zimmerman SA, Schultz WH, Ware RE (2001). Hydroxyurea therapy for pediatric patients with HbSC disease. J Pediatr Hematol Oncol.

[33] Yates AM, Dedeken L, Smeltzer MP, Lebensburger JD, Wang WC, Robitaille N (2013). Hydroxyurea treatment of children with HbSC disease. Pediatr Blood Cancer.

[34] Opoka RO, Ndugwa CM, Latham TS (2017). Novel use Of Hydroxyurea in an African Region with Malaria (NOHARM): a trial for children with sickle cell anemia. Blood.

[35] Tshilolo L, Tomlinson G, Williams TN (2019). Hydroxyurea for children with sickle cell anemia in sub-Saharan Africa. N Engl J Med.

[36] John CC, Opoka RO, Latham TS (2020). Hydroxyurea dose escalation for sickle cell anemia in sub-Saharan Africa. N Engl J Med.

